# Exploring member’s knowledge sharing intention in online health communities: The effects of social support and overload

**DOI:** 10.1371/journal.pone.0265628

**Published:** 2022-03-24

**Authors:** Chiahui Yen

**Affiliations:** Department of International Business, Ming Chuan University, Taipei, Taiwan; Fu Jen Catholic University, TAIWAN

## Abstract

This study explores the determinants of members’ participation intention in online health communities (OHC) from both the facilitators and barriers points of view. From the facilitators perspective, each member’s subjective well-being plays a crucial role in sharing intention. On the other hand, from the barriers point of view, social network site exhaustion would negatively affect. The survey was conducted on two online support groups, including parents of children with autism spectrum disorder and caregivers of dementia disease. This study collected 330 questionnaires from social network sites to examine the research model. The results showed that social support positively affects members’ self-efficacy; in turn, self-efficacy has a positive effect on subjective well-being. Overload has an impact on psychological distress. Moreover, members’ subjective well-being determined their knowledge sharing intention.

## 1. Introduction

*Online health communities (OHC)* are becoming valuable platforms for users to communicate and find support on social network sites (SNS). The importance of OHC is evidenced by their popularity and the significant impact they have on the lives of their members. For instance, most members believe the Internet is an excellent place to search for medical information, and they actually get helpful information and advice [1]. Further, OHC allows members to exchange information via the Internet with like-minded people with the same health conditions and similar experiences in order to exchange social support [2–5]. These developments give rise to an important question: *Is it possible to encourage greater participation and knowledge sharing intention among community members who interact through OHC*?

There has been a growing interest in examining the factors that support or hinder one’s knowledge sharing behavior in the OHC [6–8]. Knowledge sharing refers to individuals transferring their acquired knowledge to other organization members. Previous studies have highlighted various factors that influence individuals’ willingness to share knowledge, such as costs and benefits, incentive systems, extrinsic and intrinsic motivations, and organizational climate [9–11]. Recently, some studies have investigated the various factors that influence members’ knowledge sharing intention in an online health context. Goh, Gao et al. [12], Jin, Li et al. [13], Chung, Nam et al. [14], as well as Deng and Liu [15] suggested OHC create social value through social-related perspectives, such as social support, social identification, and social capital. Some researchers viewed knowledge sharing as an exchange behavior and then explored the antecedents of sharing intention from benefit and cost factors [16, 17]. However, still few studies examined them from both facilitators and barriers perspectives. Therefore, a comprehensive understanding of the knowledge sharing motivations in the context of OHC is essential.

This study aims to understand factors that influence members’ knowledge sharing intention in OHC from facilitators and barriers viewpoints. Moreover, this study focuses on *subjective well-being* as facilitators and *SNS exhaustion* as barriers to sharing intention. Accordingly, we investigate the effects of *social support* on self-efficacy and subjective well-being, while we emphasize the effect of *overload* on psychological distress and SNS exhaustion.

On the one hand, *subjective well-being* focuses on hedonic perspectives, in terms of positive affect, such as happiness and life satisfaction. Subjective well-being also could reduce negative affects, such as stress and anxiety. The organizational behavior literature indicates that subjective well-being significantly increases organizational commitment and positive behavior. In particular, knowledge sharing behavior SNS can benefit OHC members, who experience more social support and have more subjective well-being. Further, *social support* is viewed as an exchange of resources between at least two individuals that is perceived by the provider or recipient to enhance the welfare of the recipient [18]. Social support has been identified as an essential buffer of mental health [19], and as any process through which social relationships might promote health and well-being [20, 21]. The concept and the related theory of social support have been studied for decades, and researchers have endeavored to theorize the social support function and examine the role of social relationships and embedded social support in mediating personal life stress [22].

On the other hand, members may be drawn into exhausting social situations. Studies have found many social relations enabled in SNS might cause users to feel they are giving too many messages to respond to their social networks. *SNS exhaustion* is the psychological and behavioral consequence of excessive use of social media, which leads to low levels of satisfaction. This fatigue phenomenon also reflects the psychological response of individuals to the stress-coping conditions induced by SNS use [23, 24]. In this situation, the overload phenomenon might explain why an increasing number of SNS users feel exhausted while using social media [24, 25]. Addressing this phenomenon, some researchers investigated how overload factors influence SNS exhaustion and continuous usage of SNS [26, 27].

Since OHC are social entities comprised of people and their relationships, their success depends on members’ behaviors that benefit the community as a whole. However, both patients and caregivers may not always be motivated to join support communities and share their experiences with others in difficult situations. For patients, it is not easy to face the disease and talk about privacy. For caregivers, caring for a loved one can be very rewarding, but it also involves many stressors. What motivates individuals to involve OHC and offer support to other members? More significant for the facilitators of participation, however, what exactly are the obstacles for the individual to sharing knowledge in OHC? Although prior Information Systems studies, as well as public health and medical studies, have focused on the effects of social support for caregivers/patients in OHC [28–31], there have been still existed several research gaps.

First, there is no study to date that attempts to integrate the effects of both social support and overload on members’ knowledge sharing intention. Despite the fact that considerable scholarly attention has focused on the positive psychological status of online social support on behavior outcome [22, 32], there has been little research to raise negative factors that hinder knowledge sharing intention in OHC setting. In particular, this study highlights the force of overload factors on negative psychological status and behavior. Studies belonging to this stream were still limited, and several questions were not fully addressed.

Second, previous research related to OHC mainly focused on the information provided by patients and mechanisms of community operation, while content analysis was usually used to code messages [2, 33]. However, to date, quantitative literature on the multidimensional effect of social support is lacking despite results in qualitative studies from support communities that have consistently indicated how social support constantly complicates people’s everyday lives. Therefore, the present study adopts a questionnaire survey to understand the phenomenon of participation intention in OHC.

To fill the aforementioned gaps, this study proposes that members’ knowledge sharing intention is derived from two forces in terms of increasing *subjective well-being* and reducing *SNS exhaustion*. That is to say, and this study focuses on social support factors as facilitators and overload factors as barriers to their sharing intention.

## 2. Theoretical background and research hypotheses

### 2.1 Theoretical background and research model

Social support can be defined as “functions performed for a distressed individual by significant others,” and the provision of social support can be conceptualized as support providers’ active participation in receivers’ stress management efforts [34]. Prior research has shown that social support has positive effects for individuals, such as reducing stress, getting benefits, and improving health. Likewise, this study expects that individuals’ perceived online social support will positively affect their self-efficacy [35].

According to Social cognitive theory, self-efficacy denotes “people’s beliefs about their capabilities to produce designated levels of performance that exercise influence over events that affect their lives” [36]. More recently, the concept of self-efficacy has been applied to the organization or SNS context to validate the effect of personal efficacy belief in cognitive well-being [37, 38] Park, Sarnikar et al. [39] found that facilitated self-efficacy messages affect helpfulness, goal-settings, and health resilience in OHC.

The literature on subjective well-being includes happiness, life satisfaction, and positive affect [40]. Prior research has examined the relationship between the use of SNS and individuals’ well-being [41, 42], which have demonstrated that social tie, social support, and social identity have a positive impact on subjective well-being [43]. Moreover, people with a high degree of subjective well-being increase an incremental effect on knowledge sharing behavior [44]. When an individual’s well-being increases, it can be seen as a positive behavior that promotes helping others and sharing knowledge through reciprocal adaptation.

Although OHC offers more functions to allow members to express their experience and comment, knowledge contribution in OHC still seems complicated. Members join OHC to seek friendship, find social support, and solve problems. However, there is an ongoing debate about whether SNS strengthens or erodes social relationships [45–47]. The over-integration of social media into daily routines invades people’s daily lives as large social networks bring uncontrolled requests for information, communication and social support, leaving them in an exhausting situation [48]. Under the circumstances, it is essential to explain the inhibitors of members’ sharing intention.

Psychological distress is used to describe unpleasant feelings or emotions that impact an individual’s level of functioning and interfere with one’s activities of daily living. Psychological distress can lead to an individual’s negative perception of the environment, others, and self. The manifestations of psychological distress reveal states such as anxiety, depression, and behavioral or emotional outbursts [49] Past research has shown that social support and positive interactions on SNS are consistently associated with lower levels of depression and anxiety. In comparison, low social support and negative interactions on SNS are associated with higher levels of psychological distress and anxiety [50].

In everyday life, overload in the social media environment is becoming increasingly evident. This issue is frequently discussed in the fields of mass media, information technology, and social psychology [23, 24, 48, 51, 52]. Taking social media communication on the Internet as an example, people find it difficult to process the plethora of media messages they receive because it requires a sustained cognitive effort and the capacity of these resources is limited [53]. Researchers believe that the overload caused by SNS use can negatively affect users’ mental health. With the increasing use of SNS, the effects of this can backfire and cause problems, including Internet addiction and cognitive load. Consequently, this trend has led to more research on the discontinuous use of SNS, which is driven by different factors compared with continuous use [25]. The above studies shed light on the negative side of SNS use and highlight the critical role of stressful experiences in terms of overload in inducing discontinuance behavior [52].

**Fig 1:**
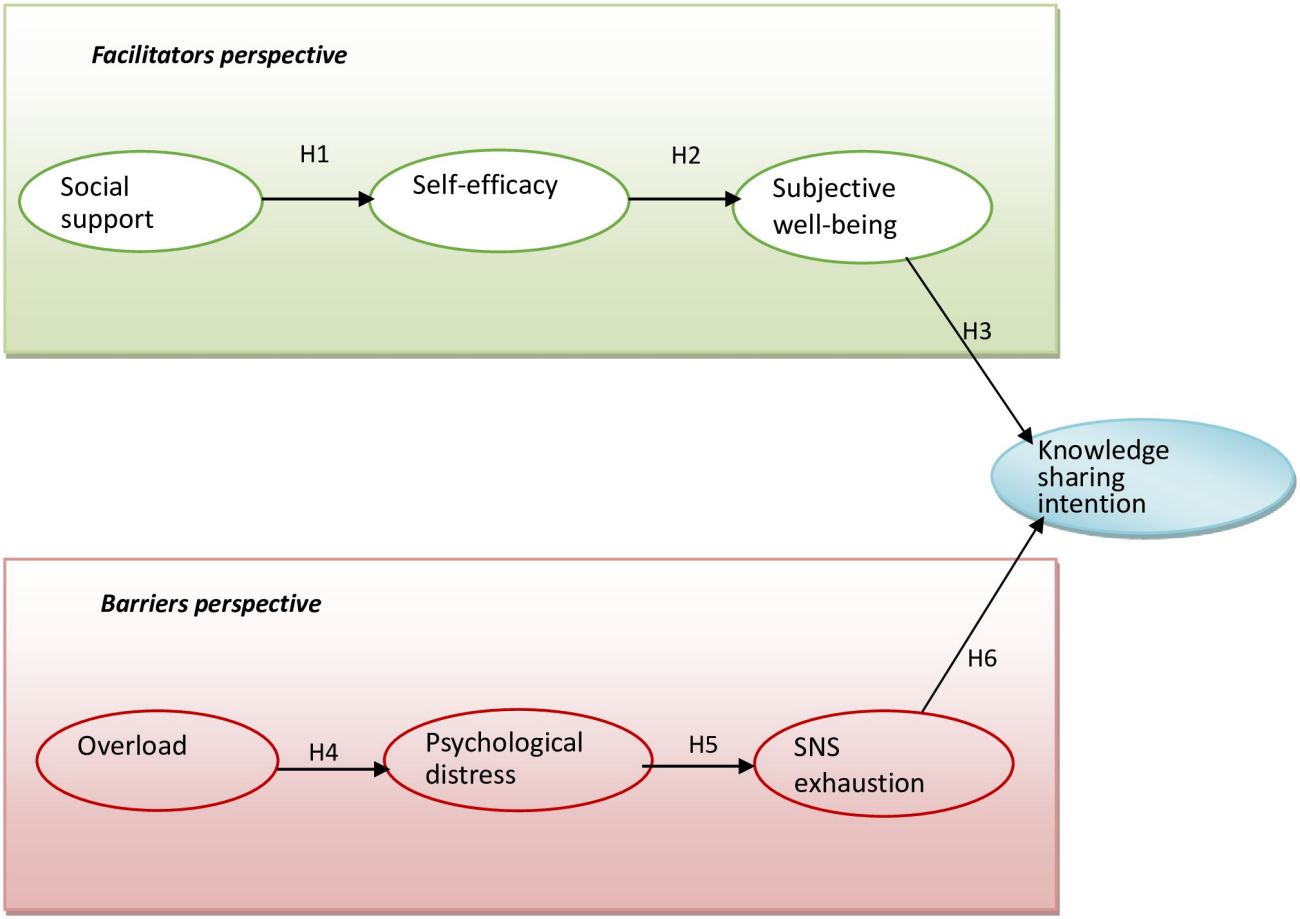
Research model.

This study attempts to discuss members’ knowledge sharing intention in OHC from facilitators and barriers perspectives. The research model is shown in Fig 1. We hypothesize that receiving online social support enhances individuals’ self-efficacy to support the community, positively promoting the knowledge sharing intention. Individuals have too much overload to cope with and then occur psychological distress, negatively promoting the willingness to share knowledge with others.

### 2.2 Facilitator dimension

*Social support* can be defined as aid and assistance exchanged through social relationships and interpersonal transactions [54]. In general, social support concerns supportive interactions embedded within interpersonal relationships [22, 55]. Bandura [56] argued that self-efficacy and outcome expectations influence behavior, with higher self-efficacy and positive expected outcomes promoting behavior change, whereas negative expectations and lower self-efficacy inhibit behavior. Moreover, members can discuss with people, gain new knowledge about the disease, and have a better understanding of professional medication or treatment, which is social support from OHC. As a result, members become confident in dealing with various types of health information, and then their self-efficacy increases. Past studies show that informational support and tangible support of social value are hypothesized to exert positive effects on self-efficacy [15]. Hence, we propose the following hypothesis:

**H1**: Social support has a positive effect on members’ self-efficacy toward OHC.

The concept of welling-being consists of general positive affect and emotional ties [49]. Several shreds of evidence show that knowledge self-efficacy is positively associated with knowledge sharing [57, 58]. Past research has shown that users with higher self-efficacy perceive information shared through SNS as more trustworthy than those with lower self-efficacy [59]. Members were concerned that the health knowledge they provided might be irrelevant or unreliable. Hence, members with high knowledge self-efficacy perceived their responses as helpful and felt they were among the few respondents who could provide valuable knowledge to patients. When the member has a high level of self-efficacy, it will result in positive behavior and then has a high level of subjective well-being. Therefore,

**H2**: Members’ self-efficacy has a positive effect on their subjective well-being toward OHC.
**H3**: Member’s subjective well-being has a positive effect on their knowledge sharing intention.

### 2.3 Barriers dimension

SNS overload includes technology overload, information overload, social overload, and emotional overload. First, the concept of technology overload is derived from technostress. Technology overload reflects SNS users’ perception of technology features, and it is framed as the SNS platform providing more functionality than users need. Second, information overload is due to the large amount of information generated by SNS, which exceeds the capacity of what one member can handle. People have limited cognitive comprehension and message processing abilities. When the received information exceeds the processing capacity, users may overload themselves with too much information, which leads to impairments such as reduced information processing ability and slower cognitive reflection [60]. Third, social overload is a situation where users need to provide too much social support to other individuals embedded in their social networks, and this situation will lead to pressure on themselves [24] Although social media has brought much convenience to people, it has also led to quite a few annoyances [27]. Members have to deal with many online activities from others when they log on to social media. Out of duty to their friends, they may force themselves to deal with a large number of social requests for the sympathy of others. Finally, the condition of members’ perceived emotional overload is related to their exhaustion. SNS exhaustion refers to a person’s aversive and involuntary emotional reaction to tiring situations associated with the use of SNS [24, 51]. Although OHC gratifies the member’s needs, it may cause SNS exhaustion that is reflected by negative feelings and reduction in interest. Therefore, we put forward the following hypothesis:

**H4**: Member’s perceived overload of OHC has a positive effect on psychological distress.

SNS exhaustion could be viewed as social network fatigue which is the adverse emotional reaction to social network activities, such as lower interest, fatigue, tiredness, boredom, and burnout [25]. When members develop SNS fatigue, they avoid this uncomfortable and frustrating situation and thus reduce the frequency and duration of SNS use or even more completely stop using SNS or quit OHC [24]. We argued that SNS exhaustion is negatively influences continuance intention [51]. Therefore,

**H5**: Member’s psychological distress of OHC has a positive effect on SNS exhaustion.
**H6**: Member’s SNS exhaustion toward OHC has a negative effect on knowledge sharing intention.

## 3. Method

### 3.1 Research design principles and sample collection

This study involved two OHC as research targets, caregivers of autism patient support community and dementia support community. The survey was conducted among members of OHC. A link to the web questionnaire was posted on the forums. The first page of the questionnaire addresses the purpose of this study, the length of the questionnaire, the incentive, and ensures confidentiality. The respondents were instructed to answer all the questions based on their experience in this OHC. Three hundred thirty samples (n = 330) were gathered in July 2020. 72% are female, 49% are 31–41 years old, more than 45% have a high school degree, and most are family caregivers (89%).

The first survey target is “Good luck people with dementia community” which is a small forum for family caregivers of dementia in facebook. It is a private group with about 300 registered members. Every resisted member must elaborate patient’s privacy detail, including real name, medical records, and caregiver’s description data. Dementia is one of the significant issues among older people globally, which brings deterioration in cognitive function and activities of daily living [61, 62]. Worldwide, 47.5 million people have dementia, and there are 7.7 million new cases every year. Dementia has physical, psychological, and social stress on caregivers, families and society [63]. There is no therapy or medicine currently available to cure dementia or modify its progressive course, and it affects whole families overwhelmingly. The second OHC is from families with Autism spectrum disorder (ASD) members. Complex requirements of a person with an ASD can put families under tremendous stress, such as emotional, financial, and physical responses. However, giving family support and finding long-term care can help people with ASD improve their quality of life. It is critical to find health care and family providers who are comfortable with persons who have an ASD. The survey was conducted on “We have special kid” an online community in LINE with 495 registered members.

### 3.2 Procedures and measures

We used multiple items based on five-point Likert scales ranging from strongly disagree (1) to strong agree (5) for all constructs strongly. Whenever possible, we adapted scales from existing research. In order to assure the quality of experiment procedures, this questionnaire was consulted with two psychologists, one health care professional and one special education professional. Items of social support are adapted from Cutrona and Suhr [64]. Items of self-efficacy are from Lin, Hsu et al. [65] and Chiu, Huang et al. [21]. Subjective well-being is measured using items adapted from Brooks [66]. Knowledge sharing intention was measured using items adapted from Hsu, Chang et al. [67] and Bock, Zmud et al. [9]. Items for overload are from Zhang, Zhao et al. [25] and Yang and Lin [60]. SNS exhaustion is measured with items adapted from Maier, Laumer et al. [24]. Items for measuring psychological distress are modified from Veit and Ware [49]. Table 1 lists measurement items.

**Table 1:** Measurement items.

Items
Social support (SS)
Members in the online support community compliment my ability to deal with patient’s symptoms.
Members agree with how I dealt with my problems.
Members of the online support community are on my side.
Members comfort and encourage me when I face difficulties.
Self-efficacy (SE)
I can solve patient’s problems if I try hard enough.
When I am confronted with patient’s problems, I can usually find solutions.
I have set some definite goals to improve patient’s symptoms.
Subjective well-being (WB)
In most ways, my life is close to my ideal.
I am satisfied with my life.
So far, I have gotten the important things I want in life.
Knowledge sharing intention (KS)
I try to share my medical-related experience with other members.
I intend to share my experience with other members more frequently in the future.
I will share my knowledge with other members in the future.
Overload
There are too much information on this group so that I am burdened in handling it.
I am often too concerned for my friends in this group.
I find that I am overwhelmed by the amount of information I have to process on a daily basis.
Psychological distress
I feel anxious.
I feel depressed.
I lost behavior control.
I lost emotional control.
SNS exhaustion
I feel drained from activities that require me to use SNS.
I feel tired from my SNS activities.
I feel burned out from my SNS activities.

## 4. Data analysis and results

### 4.1 Data analysis

A partial least squares structural equation modeling (PLS-SEM) approach, supported by SmartPLS, was utilized in this study in order to analyze the survey results [68]. Reliability and validity are the two most essential and fundamental features in evaluating the measurement model. The results found that composite reliability and Cronbach’s alpha figures were above 0.7, as shown in Table 2, exceeding the suggested level.

**Table 2:** Results of reliability and convergent validity.

Construct	Items and Factor loading	Composite reliability	Cronbach’s Alpha
Social support (SS)	SS1 = 0.72 SS2 = 0.87 SS3 = 0.91 SS4 = 0.88	0.91	0.87
Self-efficacy (SE)	SE1 = 0.87 SE2 = 0.77 SE3 = 0.79	0.85	0.75
Knowledge-sharing intention (IN)	IN1 = 0.94 IN2 = 0.95 IN3 = 0.81	0.93	0.89
Subjective well-being (WB)	WB1 = 0.91 WB2 = 0.94 WB3 = 0.93	0.95	0.92
Overload (SO)	SO1 = 0.93 SO 2 = 0.82 SO 3 = 0.81	0.89	0.82
Psychological distress (PD)	PD1 = 0.86 PD 2 = 0.88 PD 3 = 0.84 PD4 = 0.91	0.93	0.90
SNS exhaustion (EX)	EX1 = 0.91 EX 2 = 0.92 EX 3 = 0.95	0.95	0.86

The average variance extracted (AVE), typically used to assess convergent validity [69], indicates how much of the latent variable can explain the indicators’ variance. An AVE larger than 0.5 has been suggested to provide empirical evidence for convergent validity. In our study, AVE values were more than 0.72, and convergent validity was determined adequately. Regarding discriminant validity, a novel approach for assessing it would be the heterotrait-monotrait ratio of correlations (HTMT) [70]. Discriminant validity entails that two latent variables representing two different theoretical concepts are statistically different. To obtain empirical evidence for discriminant validity, the HTMT should be lower than 0.85 [71]. In our study, all HTMT values were below the recommended threshold, as presented in Table 3, and demonstrated adequate discriminant validity.

**Table 3:** Results of AVE and HTMT.

	AVE	PD	EX	SE	SO	SS	WB
SNS exhaustion	0.86	0.11					
Self-efficacy	0.66	0.56	0.08				
Overload	0.73	0.25	0.64	0.22			
Social support	0.72	0.58	0.05	0.54	0.23		
Subjective well-being	0.86	0.78	0.09	0.62	0.17	0.70	
Knowledge sharing intention	0.82	0.68	0.07	0.49	0.15	0.55	0.76

### 4.2 Model testing results

**Fig 2:**
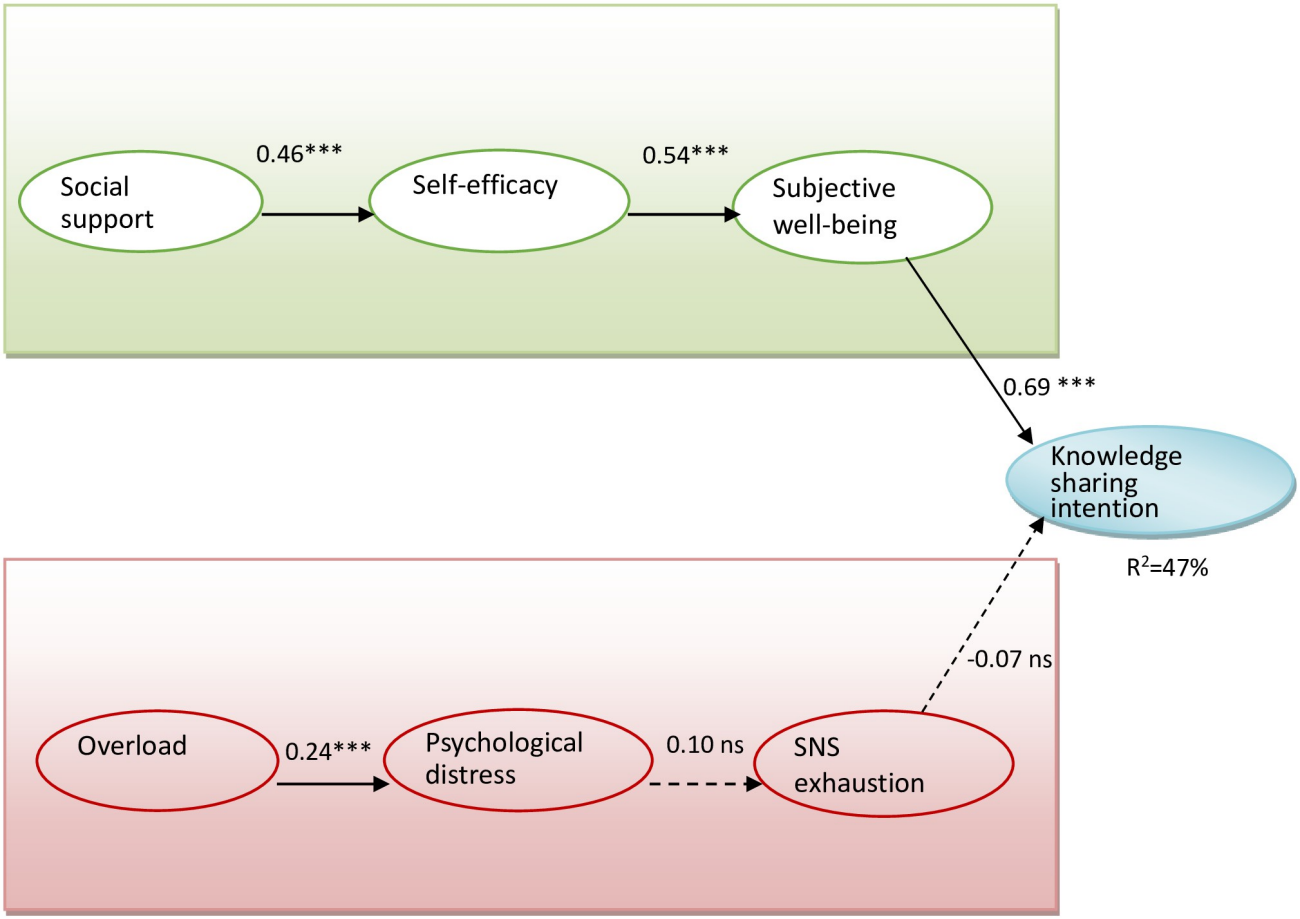
Path analysis of this study. ***p<0.001 ns. Non-Significant.

H1, H2, H3, and H4 displayed P-values smaller than 0.001, and Fig 2 showed path analysis results. The significance of all paths was assessed through 2000 bootstrap runs. Social support had significant effects on self-efficacy (β = 0.46, t = 10.59), meaning that H1 was verified. Self-efficacy positively influenced subjective well-being (β = 0.54, t = 12.55), and H2 was supported. H3 argued that subjective well-being would predict knowledge sharing intention (β = 0.69, t = 21.42). Therefore, H3 was supported. Overload significantly affected psychological distress (β = 0.24, t = 4.62). H4 was supported as well. However, the path coefficients suggested that psychological distress does not significantly impact SNS exhaustion (β = 0.10, t = 1.7), indicating that H5 was not supported. SNS exhaustion did not predict knowledge sharing intention (β = -0.07, t = 1.67). H6 was not supported, either. Overall, the results show that the research model can explain 47.1% of the variance of knowledge sharing intention.

## 5. Findings and implications

### 5.1 Findings and discussion

The overall findings verify the theoretical framework, and this paper has made several critical contributions to research. First, the results demonstrate that social support is significantly related to self-efficacy. OHC can be a helpful platform for exchanging information with people facing similar health concerns. When members receive information from a support group, they will have more confidence when dealing with health issues and problems relating to the patient. As they believe that they can learn and improve, self-efficacy will thus increase.

Second, we also highlighted the role of self-efficacy as the dominant force in shaping members’ subjective well-being in OHC. This finding suggests that when members’ self-efficacy increases, they have a more positive attitude toward their lives and help other members. Furthermore, self-efficacy attracts interest increasingly as a protective variable regarding the caregiver’s burden. Consequently, self-efficacy, as an individual’s self-concept goal, emerges as an overarching objective to improve subjective well-being [58].

Third, the results validate the role of subjective well-being as a positive factor through which to enhance caregivers’ knowledge contribution intentions. People join online support groups because they have to accept the reality of the situation and find better solutions. The health community involvement of individuals is motivated by various aspects, and they share knowledge with others who have the same problems in OHC while also avoiding negative situations.

However, this paper found that psychological distress does not have a significant effect on SNS exhaustion. A possible explanation for this is that community members are under a lot of pressure, and there are other ways to relieve that pressure. Therefore, psychological distress does not cause burnout and boredom.

### 5.2 Implications

This study proved that subject well-being and SNS exhaustion affect knowledge sharing intention from both aspects. Within the context of OHC, the focus of most studies was to identify facilitators of members’ participation intention. This study showed that participation in OHC provided members with some positive psychological benefits and control in managing the health problems of those in their care, as well as an enhanced sense of satisfaction in helping others. Moreover, OHC could generate substantial social value for participants [12], and even improve their social well-being and quality of life. Members discuss their problems with others and listen to other members’ difficulties, and they are getting support and being able to help others through this channel. Members might find out that he is not the only one suffering and could feel easier. Other member’s experience and knowledge can be invaluable, especially as they are caring for someone with the same illness. This study has proved that OHC has the features necessary to have positive psychological effects. Hence, OHC is a valuable platform to receive social support and share health knowledge, one core question for researchers and practitioners in understanding the factors that can facilitate or inhibit members’ participation intention in OHC.

## Supporting information

S1 Data (CSV)
